# Joint Statement of the Korean Society for Preventive Medicine and the Korean Society of Epidemiology on the response to the COVID-19 outbreak

**DOI:** 10.4178/epih.e2020019

**Published:** 2020-04-29

**As concerns over coronavirus disease 2019 (COVID-19) continue to grow, unnecessary overreactions have occurred. In times like these, we need the cooperation and support of our citizens.**

COVID-19 has been spreading around the world. In Korea, since the first confirmed case, the number of patients with COVID-19 has increased to 27 over the last three weeks. The World Health Organization has declared it the sixth ‘Public Health Emergency of International Concern (PHEIC)’ and has called for joint international action. The Korean government has already raised our national crisis level to “alert.” However, with signs that wider community spread is imminent, an upgrade to “severe”, the highest crisis management response level, cannot be ruled out. It is challenging to manage this crisis only with the efforts of the authorities and medical institutions centered on the Korean Centers for Disease Control and Prevention (KCDC). Social cooperation from the general population is urgently needed.

At the time of the severe acute respiratory syndrome (SARS) epidemic in 2003, there were no cases found in Korea. This feat was recognized by the World Health Organization as the best defense example by a country. Experts believe that the emergency response system to combat COVID-19 in Korea is on par with other countries. It appears that the biggest obstacles in overcoming this crisis are fake news, misinformation, excessive anxiety and agitation, non-experts making claims with little evidence, stigma against patients, and a lack of voluntary cooperation from some people with symptoms or those that are under epidemiological investigation.

More time and effort are needed to fully identify COVID-19. However, a consensus among experts has been reached on what actions are required in order to end the outbreak as soon as possible. We ask for citizens to trust the expertise of the authorities and cooperate with their efforts to combat the spread of COVID-19. The Korean Society for Preventive Medicine and the Korean Society for Epidemiology, as the representative professional societies in the public health sector, would like to propose several actions for citizens based on our scholarly expertise.

First, please trust and follow the information that authorities and experts provide through official media outlets. Unproven information has been appearing online, making citizens anxious. Such information has the potential to speed the spread of the outbreak by undermining the competence of defense authorities and experts.

Second, please do not promote excessive anxiety or overreaction. Closing schools and shops near where confirmed patients have been does not affect public health. Instead, it causes unnecessary social costs due to the fear and stigma it creates. Despite the spread of the outbreak around the world, there are still few confirmed cases in countries other than China. Even in China, the fatality rate in regions outside of Wuhan and Hubei is 0.16%. This is much lower than SARS which was 9.6% and Middle East Respiratory Syndrome (MERS) which was 34.4%. Of the 318 patients (as of 9th February 2020) confirmed to have COVID-19 outside of China, there have been only two confirmed deaths. In Korea, there are 27 confirmed patients, but so far, no deaths have occurred. Some of the early confirmed patients are now considered cured. The United States of America Centers for Disease Control and Prevention (CDC) has not restricted United States citizens from traveling to Korea. The CDC still views Korea as a “first grade” safe state, which is the same as prior to the outbreak.

Third, please do not be deceived by solutions from non-experts. Handwashing with soap, coughing etiquette, mask use by those patients with a cough, visiting a screening clinic promptly, and honest disclosure of overseas travel history are proven prevention measures. Proper disinfection of the facilities and workplaces visited by confirmed patients is sufficient, with long-term closures being unnecessary. Restricting the entry of foreigners to Korea needs to be approached carefully and should take into account reciprocal agreements between countries. Garlic intake, use of anti-inflammatory analgesic ointments, and avoiding imported foods from China have no effect and can result in more serious side effects.

Fourth, stigma against patients should be avoided. This violates human dignity and rights. It also makes rapid diagnoses and patient management more challenging. Patients will circumvent the authorities in situations where they are blamed, unconditionally quarantined, or prevented entry to facilities due to having a fever or cough. Additionally, closing facilities where patients have visited can be harmful. The success of infectious disease prevention depends on protecting human rights, not exclusion and discrimination. It is clear that the current situation is a public health crisis, but we do not consider it a threat to our daily lives. Irrational fears should not govern our daily lives, with unnecessary overreactions causing further damage.

Fifth, for citizens who are suspected of having COVID-19 after being in contact with a confirmed patient, visiting China, or a country with the epidemic, please do not delay but voluntarily report to the nearest public health center and actively cooperate with the follow-up measures based on your test results. Caring for patients and people that they have come in to contact with is central to the protection measures against COVID-19. It determines the health of the patient and their family as well as the success or failure of it spreading in the community. The right to healthcare, as defined by the Constitution and the Health Care Law, should be upheld.

Finally, we would like to advise government officials that for further measures on dealing with confirmed patients, please consult the KCDC, and Ministries of Health and Welfare, Education, Public Administration, and Security, and National Defense so as to avoid unnecessary confusion. At a regional level, we suggest that local government, school boards, universities, disaster-related agencies, and civil society organizations operate regionally integrated command headquarters where they can share accurate information on local conditions and defense strategies so that their efforts can be integrated together.

10 February 2020

The Korean Society for Preventive Medicine and the Korean Society of Epidemiology Emergency Committee on the Coronavirus

Disease (COVID-19) Outbreak

