# Supplementary material for: Joint Statement of the Korean Society for Preventive Medicine and the Korean Society of Epidemiology on the response to the COVID-19 outbreak
Source: Epidemiol Health. 2020 Apr 29;42:e2020019. doi: 10.4178/epih.e2020019 (PMC7285423; doi:10.4178/epih.e2020019)
Supplement: Supplementary file 1 [file epih-42-e2020019-suppl.pdf]

Volume: 42, Article ID: e2020019
https://doi.org/10.4178/epih.e2020019

신종코로나바이러스감염증 위기 극복을 위한 대한예방의학회·한국역학회 공동 성명서

신종코로나바이러스감염증에 대한 우려가 커지면서 불필요한 과잉대응을 요구하는 목소리가 커지고 있습니다. 이럴 때일수록 차분하고 이성적인 시민들의 협력과 실천이 필요합니다.

○ 신종코로나바이러스감염증이 전 세계로 확산되고 있습니다. 국내에서도 첫 확진 사례 이후 3 주 만에 확진 환자가 27 명으로 늘었습니다. 세계보건기구에서는 여섯 번째의 '공중보건 위기상황'으로 선포하여 국제적 공동대응을 이끌고 있습니다. 한국 정부도 감염병 위기경보 수준을 진작에 '경계' 단계로 끌어올렸습니다. 하지만 지역사회 확산이 임박했다는 징후가 나타나면서, 위기경보 최고 수준인 '심각' 단계로의 격상도 배제할 수 없는 상황입니다. 질병관리본부를 중심으로 한 방역당국과 의료기관의 노력만으로는 이러한 위기를 극복하기 어렵습니다. 전 사회적인 협력과 실천이 절실합니다.

○ 2003 년 사스(SARS) 유행 당시 국내에서는 단 한 명의 확진 사례도 없었으며, 세계보건기구로부터 가장 모범적인 방역성과를 거둔 국가로 인정받기도 했습니다. 현재 신종코로나바이러스감염증 확산 저지를 위한 비상대응체계 또한 어느 국가들과 비교해도 뒤지지 않는다는 것이 전문가들의 평가입니다. 이 시점에서 신종코로나바이러스감염증 위기극복의 가장 큰 장애물은 가짜뉴스와 왜곡된 정보, 과도한 불안과 선동, 비전문가들이 쏟아내는 근거 부족의 백가쟁명식 해법, 환자와 접촉자에 대한 낙인, 역학조사 대상자들과 일부 유증상자의 자발적 협조 부족입니다.

○ 신종코로나바이러스감염증의 실체를 완벽하게 드러내기 위해서는 더 많은 시간과 노력이 필요합니다. 그러나 유행의 조기 종식을 위해 어떤 조치가 필요한지는 이미 전문가들의 합의가 도출되어 있습니다. 방역당국과 관련 전문가들의 역량과 노력을 시민들이 신뢰하고 협조해주시기를 부탁드립니다. 대한예방의학회와 한국역학회는 국내 공중보건 분야를 대표하는 전문학회로서 학회원들의 학자적 양심과 전문 지식에 기반하여 다음과 같은 사회적 협력과 시민들의 실천을 제안하고자 합니다.

첫째, 방역당국과 전문가들이 공식 매체를 통해 전달해 드리는 정확한 정보를 믿고 따라주십시오. 현재 온라인과 뉴미디어에는 검증되지 않은 자극적인 정보들이 범람하며 시민들을 불안하게 만들고 있습니다. 이는 방역당국과 전문가들의 대응역량을 분산시킴으로써 유행의 확산을 촉진시킬 가능성이 있습니다.

Volume: 42, Article ID: e2020019
https://doi.org/10.4178/epih.e2020019

둘째, 과도한 불안을 조장하거나 효과 없는 과잉대응을 조장해서는 안 됩니다. 확진자가 다녀간 지역 인근의 학교와 상점이 문을 닫는 것은 공중보건 측면에서 아무런 효과가 없습니다. 오히려 공포와 낙인 때문에 불필요한 사회적 비용만 소모하게 됩니다. 현재 유행이 전 세계로 확산되기는 했지만, 초기 방역에 실패하여 걷잡을 수 없이 지역사회로 퍼져나간 중국을 제외하면 여타 국가들에서 확진 사례 발생은 여전히 많지 않습니다. **중국에서도 우한과 후베이성을 제외한 지역의 치명률은 0.16%로 사스 9.6%, 메르스 34.4%에 비해 매우 낮습니다.** 중국 이외 국가에서 발생한 전체 환자 318 명(2020/2/9 기준) 중에서 사망건수는 2 명에 불과하 고, 우리나라의 경우는 27 명의 확진 환자가 발생되었을 뿐 사망자는 없었습니다. 이제는 초기 확진환자들 중에서 완치사례가 속속 나오고 있습니다. 미국 질병관 리본부(CDC)에서도 자국민의 우리나라 여행을 제한하지 않고 있으며, 유행 이전 과 다름없이 한국을 “1 등급”의 안전한 상태로 평가하고 있습니다.

셋째, 비전문가들의 백가쟁명식 해결책에 현혹되어서도 안 됩니다. 비누로 손씻기, 기침예절, 발열, 기침 환자의 마스크 착용, 신속한 선별진료소 방문과 해외여행력 등의 정직한 공개가 현재까지 검증된 예방수칙입니다. 확진 환자가 방문한 시설과 직장환경의 적정 소독으로 충분하며 장기간 폐쇄하는 것은 불필요하고, 외국인 입국 제한에 있어서는 국가 간 상호주의 원칙에 입각한 신중한 접근이 필요합니다. 또한 마늘 섭취, 진통소염 연고 도포, 중국산 수입식품 배척 등은 아무런 효과가 없거나 부작용이 더 큽니다.

넷째, 환자와 접촉자 등에 대한 낙인은 타인의 존엄한 인권을 침해하는 것일 뿐 아니라, 오히려 신속한 진단과 환자관리를 어렵게 만든다는 점에서 피해야 할 행동입니다. 환자를 비난하고, 접촉자를 무조건 격리하며, 발열, 기침 증상이 있다고 하여 공동시설 출입을 막거나, 전파력이 있는지를 제대로 따지지도 않고 확진 환자가 다녀간 곳을 일단 폐쇄하는 분위기에서는 당사자들이 방역당국을 피해 다니게 됩니다. **감염병 방역활동의 성패는 배제와 차별이 아니라 포용과 인권보호에 달려 있다는 것이 그동안 감염병 유행에서 얻은 보건학적 교훈입니다.** 지금은 공중보건학적 위기상황인 것은 분명하지만, 우리의 일상을 위협할 수준으로까지 진행되었다고 볼 수 없습니다. 실재하는 위협과 이에 대한 합리적 대응을 넘어 비이성적 공포로 인해 일상적 삶을 유지하지 않고, 불필요한 과잉대응으로 2 차 피해를 유발해서는 안 될 것입니다.

Volume: 42, Article ID: e2020019
https://doi.org/10.4178/epih.e2020019

다섯째, 확진 환자와 접촉하여 자가격리 대상에 해당되거나 중국 또는 유행국가를 방 문한 후 신종코로나바이러스감염증 의심증상이 있는 시민은 지체하지 마시고 보 건소에 자발적 신고를 해주시고, 검사결과에 따른 후속조치에 적극적으로 협력하 여 주시기 바랍니다. 환자와 접촉자들에 대한 관리는 신종코로나바이러스감염증 방역조치의 핵심이며, 본인은 물론 가족의 건강을 보호하고 지역사회 확산 예방 의 성패를 결정합니다. 이는 헌법과 보건의료기본법에서 규정하는 건강권을 보장 받는 길이기도 합니다.

끝으로 정부 관계자께 요청드립니다. 확진환자 방문지역에 대한 추가조치 등은 질병관리본부 와 보건복지부, 교육부(교육청), 행정안전부, 국방부 등이 상호 협의해 기본원칙과 지침을 마련 해서 불필요한 혼선을 피할 수 있게 해주십시오. 아울러 지역단위에서는 지방정부, 교육청, 대 학교, 재난 관련 각급기관, 그리고 시민사회단체들이 지역의 상황과 효과적 방역 등에 관한 정확한 정보를 공유하며 총력 방역에 의기투합할 수 있는 지역통합지휘본부를 즉각 가동하실 것을 제안합니다.

2020 년 2 월 10 일

대한예방의학회· 한국역학회 신종코로나바이러스감염증 대책위원회
